# Implementing Symptom-Based Predictive Models for Early Diagnosis of Pediatric Respiratory Viral Infections

**DOI:** 10.3390/v17040546

**Published:** 2025-04-08

**Authors:** Antoni Soriano-Arandes, Cristina Andrés, Aida Perramon-Malavez, Anna Creus-Costa, Anna Gatell, Ramona Martín-Martín, Elisabet Solà-Segura, Maria Teresa Riera-Bosch, Eduard Fernández, Mireia Biosca, Ramon Capdevila, Almudena Sánchez, Isabel Soler, Maria Chiné, Lidia Sanz, Gabriela Quezada, Sandra Pérez, Dolors Canadell, Olga Salvadó, Marisa Ridao, Imma Sau, Ma Àngels Rifà, Esperança Macià, Sílvia Burgaya-Subirana, Mònica Vila, Jorgina Vila, Asunción Mejías, Andrés Antón, Pere Soler-Palacin, Clara Prats

**Affiliations:** 1 Pediatric Infectious Diseases and Immunodeficiencies Unit, Children’s Hospital, Vall d’Hebron Barcelona Hospital Campus, 08035 Barcelona, Spain; pere.soler@vallhebron.cat; 2 Infection and Immunity in Pediatric Patients, Vall d’Hebron Research Institute, 08035 Barcelona, Spain; andres.anton@vallhebron.cat; 3 Respiratory Virus Unit, Microbiology Department, Vall d′Hebron Research Institute, Vall d′Hebron University Hospital, Vall d′Hebron Barcelona Hospital Campus, 08035 Barcelona, Spain; cristina.andresverges@vallhebron.cat (C.A.); andres.anton@vallhebron.cat (A.A.); 4 Computational Biology and Complex Systems (BIOCOM-SC) Group, Department of Physics, Barcelona School of Agri-Food and Biosystems Engineering, Universitat Politècnica de Catalunya, 08860 Castelldefels, Spain; aida.perramon@upc.edu (A.P.-M.); clara.prats@upc.edu (C.P.); 5 Department of Pediatrics, Children’s Hospital, Vall d’Hebron Barcelona Hospital Campus, 08035 Barcelona, Spain; anna.creus@vallhebron.cat (A.C.-C.); jorgina.vila@vallhebron.cat (J.V.); 6 Equip Pediatria Territorial Garraf, 08812 Barcelona, Spain; annagatellcarbo@gmail.com; 7 CAP Marià Fortuny, Reus, 43205 Tarragona, Spain; ramonamar13@yahoo.es (R.M.-M.); gquezadac76@gmail.com (G.Q.); 8 EAP Vic Nord, Vic, 08500 Barcelona, Spain; esolas.cc.ics@gencat.cat (E.S.-S.); triera.cc.ics@gencat.cat (M.T.R.-B.); efernandez.cc.ics@gencat.cat (E.F.); 9 EAP Les Borges Blanques, 25400 Lleida, Spain; mireiabiosca@gmail.com (M.B.); capbert@gmail.com (R.C.); 10 CAP Les Hortes, 08004 Barcelona, Spain; asanva@yahoo.es; 11 EAP Navàs-Balsareny, 08670 Barcelona, Spain; isolerg.cc.ics@gencat.cat; 12 CAP Almacelles, 25100 Lleida, Spain; mchine.lleida.ics@gencat.cat; 13 CAP Seròs, 25183 Lleida, Spain; sanzborrell@gmail.com; 14 CAP Barberà del Vallès, 08210 Barcelona, Spain; sandra_sag88@hotmail.com (S.P.); dcanadell03@gmail.com (D.C.); 15 CAP Llibertat, Reus, 43201 Tarragona, Spain; olgasalvadojuncosa@gmail.com; 16 EAP Sant Vicenç dels Horts, 08620 Barcelona, Spain; marisaridao@gmail.com; 17 EAP Santa Coloma de Farners, 17430 Girona, Spain; isau.girona.ics@gencat.cat; 18 CAP Tona, 08551 Barcelona, Spain; marifa.cc.ics@gencat.cat; 19 CAP Manlleu, 08560 Barcelona, Spain; emacia.cc.ics@gencat.cat (E.M.); sburgaya.cc.ics@gencat.cat (S.B.-S.); 20 EAP Horta, 08032 Barcelona, Spain; mvilad.bcn.ics@gencat.cat; 21 Department of Infectious Diseases, Diseases, St. Jude Children’s Research Hospital, Memphis, TN 38105, USA; asuncion.mejias@stjude.org

**Keywords:** pediatric care, symptom-based predictive modeling, respiratory virus infections, acute respiratory infection, triage

## Abstract

(1) Background: Respiratory viral infections, including those caused by SARS-CoV-2, respiratory syncytial virus (RSV), influenza viruses, rhinovirus, and adenovirus, are major causes of acute respiratory infections (ARIs) in children. Symptom-based predictive models are valuable tools for expediting diagnoses, particularly in primary care settings. This study assessed the effectiveness of machine learning-based models in estimating infection probabilities for these common pediatric respiratory viruses, using symptom data. (2) Methods: Data were collected from 868 children with ARI symptoms evaluated across 14 primary care centers, members of COPEDICAT (Coronavirus Pediatria Catalunya), from October 2021 to October 2023. Random forest and boosting models with 10-fold cross-validation were used, applying SMOTE-NC to address class imbalance. Model performance was evaluated via area under the curve (AUC), sensitivity, specificity, and Shapley additive explanations (SHAP) values for feature importance. (3) Results: The model performed better for RSV (AUC: 0.81, sensitivity: 0.64, specificity: 0.77) and influenza viruses (AUC: 0.71, sensitivity: 0.70, specificity: 0.59) and effectively ruled out SARS-CoV-2 based on symptom absence, such as crackles and wheezing. Predictive performance was lower for non-enveloped viruses like rhinovirus and adenovirus, due to their nonspecific symptom profiles. SHAP analysis identified key symptoms patterns for each virus. (4) Conclusions: The study demonstrated that symptom-based predictive models effectively identify pediatric respiratory infections, with notable accuracy for those caused by RSV, SARS-CoV-2, and influenza viruses.

## 1. Introduction

Respiratory viral infections are a predominant cause of acute respiratory infections (ARIs), particularly during peak seasons when several pathogens circulate simultaneously [1]. This is remarkably significant in children, whose underdeveloped immune system leads to specific vulnerabilities and responses to infections [2]. ARIs remain the most common cause of illnesses, hospitalizations, and mortality in the pediatric population, leading to a considerable economic burden on families and society [3]. Regarding viruses, these are categorized into families based on their genetic composition [ribonucleic acid (RNA) or deoxyribonucleic (DNA)], structural characteristics, and replication strategies [4]. RNA viruses are distinguished by their remarkable genetic diversity, a consequence of their rapid mutation rates and distinctive replication mechanisms. This capacity for rapid adaptation to new hosts, evasion of immune responses, and development of resistance to antiviral drugs is a consequence of this genetic variability [5]. Among the most prevalent viruses affecting the pediatric population are RNA viruses such as SARS-CoV-2, respiratory syncytial virus (RSV), influenza virus, rhinovirus, and one DNA adenovirus. These viral infections often exhibit overlapping seasonal patterns and symptoms—such as fever, cough, or wheezing—making clinical diagnosis particularly challenging if targeted testing is not performed [6].

During the COVID-19 pandemic, the strain placed on healthcare systems made it especially difficult to provide comprehensive diagnostic testing for all symptomatic patients [7]. Children were particularly impacted, as they often exhibited mild or atypical symptoms [8]. In this context, given the similar clinical presentation of different respiratory viral infections, symptom-based predictive models emerged as a promising approach to expedite the diagnostic process in the clinical setting. These models function as pre-diagnostic tools, helping clinicians assess the likelihood of specific infections even before confirmatory testing is available [9]. By predicting the most likely pathogen based on symptom presentation, such models enable informed clinical decisions, even in the absence of immediate diagnostic confirmation [10,11]. Nevertheless, these tools are mainly designed for use in emergency settings rather than in primary care [12], where the immediate and accurate identification of the causative pathogen can improve patient outcomes and reduce healthcare costs [13].

This study aims to assess the effectiveness of a previously validated symptom-based predictive model for pediatric SARS-CoV-2 [14] in determining the risk of infection for five common pediatric respiratory viruses (SARS-CoV-2, RSV, influenza viruses, rhinovirus, and adenovirus). Additionally, it explores the distinct symptom signatures associated with each viral infection, providing insights into their diagnostic utility.

## 2. Materials and Methods

### 2.1. Study Design and Sample Collection

The study was conducted across 14 primary care centers within the COPEDICAT (Coronavirus Pediatria Catalunya) network from October 2021 to October 2023. Following a structured sampling protocol, we randomly enrolled the first two patients with a suspected ARI per week from each center and obtained a mid-turbinate swab sample from each participant. The samples were sent to the reference laboratory (Hospital Vall d’Hebron) exclusively for research purposes.

### 2.2. Inclusion Criteria

The study included children based on three clinical criteria: (1) presence of bronchiolitis, defined as the first episode of respiratory infection in children under 2 years, presenting with fever, rhinorrhea, cough, and crackles or wheezing on lung auscultation; (2) presence of fever, defined by body temperature of 38 °C or higher lasting more than 24 h, in previously healthy children aged 3 months to 2 years, with a stable general condition and normal physical examination; (3) presence of influenza-like illness, indicated by a body temperature of 38 °C or higher, along with one or more additional symptoms, such as myalgia, headache, general malaise, gastrointestinal symptoms, cough, rhinorrhea, and/or odynophagia.

### 2.3. Ethical Considerations

The study was approved by the ethical committee of the coordinating center with the expedient number PR(AMI)40/2021, approved on 26 March 2021. All procedures followed the ethical standards outlined in the Declaration of Helsinki and Good Clinical Practice guidelines. Informed consent was obtained from all the legal guardian(s) of the subjects included in the study.

### 2.4. Data Collection and Processing

Clinical signs and symptoms (Table S1) were collected from the medical records of each participant into a study-designed secured database (Redcap^®^, Vancouver, BC Canada). The data were then curated to ensure accuracy, including correcting inconsistencies and addressing missing data with automated machine learning techniques. Symptoms with less than 25% missing values were included, while those exceeding this threshold were removed (Figure S1). The imputation used scikit-learn’s IterativeImputer, iterating over three rounds with five neighbors, assuming feature ordinality, to enhance data completeness. A composite binary variable indicated the presence of a specific respiratory virus (SARS-CoV-2, RSV, influenza A-B, rhinovirus, adenovirus), marking patients positive if they had a confirmatory PCR, antigen, or lab test. The influenza strains were consolidated into “Flu (A + B)” due to the limited number of samples, and each virus was modeled independently due to the low prevalence of co-infections (Figure S2).

### 2.5. Modeling Approach

For each virus, the dataset was divided into training and testing sets using 10-fold stratified cross-validation to preserve the proportion of positive and negative cases. Two machine learning models, random forest and boosting, were employed to predict the likelihood of a positive diagnosis. The data were split into two subsets: 30% for training or derivation and 70% for validation. To address class imbalance, the SMOTE-NC algorithm was applied during training, balancing both categorical and numerical features without affecting the test set. Age was calculated by subtracting the date of symptom onset from the birth date. Each model underwent hyperparameter tuning via grid search, optimizing the parameters based on the highest AUC (area under the ROC curve) on the test set for enhanced performance. Metrics such as AUC, accuracy, kappa, sensitivity, specificity, positive/negative predictive value, prevalence, detection rate, detection prevalence, and balanced accuracy were calculated for each viral infection, as detailed in the Supplementary Materials.

### 2.6. SHAP Value Analysis

Following model training and selection, SHAP (Shapley additive explanations) values were calculated for each model to assess the marginal contribution of individual symptoms to the prediction of a positive outcome. For each virus, SHAP bar plots and beeswarm plots were generated to visualize the importance of each feature in predicting that viral infection.

### 2.7. Software and Statistical Analysis

All data analysis and machine learning processes were performed using R software (version 4.4.1). A significance level of 5% was used for all statistical tests, ensuring that the results were statistically robust.

## 3. Results

### 3.1. Dataset of the Symptoms and Respiratory Viral Infections Present in the Population

We included in the analysis detailed clinical data from 868 children with a viral ARI. Among the confirmed respiratory viral infections, influenza (A + B) and RSV infections were the most prevalent, with 39.10% (*n* = 389) and 15.48% (*n* = 154) positive cases, respectively. In contrast, rhinovirus and SARS-CoV-2 infections were less common, affecting 10.35% and 9.55% of the population, respectively. The range of signs and symptoms and infection statuses used for training and evaluating the predictive model is summarized in Table S1. Fever (90.05%), cough (82.11%), and nasal congestion (81.21%) were widely reported, whereas more severe clinical presentations such as seizures (0.20%) and apnea or shock (0.00%) were rare or not identified.

### 3.2. Symptom-Based Model Performance Accuracy in the Detection of Respiratory Viruses

The symptom-based model for detecting respiratory viruses (Table 1) demonstrated solid performance across the different pathogens studied, with RSV detection achieving the highest AUC (0.81), reflecting a strong balance between sensitivity (0.64) and specificity (0.77). Also, RSV detection had the highest balanced accuracy (0.71), reflecting a superior diagnostic capability compared to that for the other viruses. COVID-19 and influenza detection also showed solid results, with AUCs of 0.71 and 0.70, respectively, translating to balanced accuracy scores of 0.64 for COVID-19 and 0.65 for influenza, indicating an acceptable and stable diagnostic performance for both infections. Although the sensitivity and specificity of COVID-19 detection (both at 0.64) were not as high as those of RSV detection, the model still achieved suitable diagnostic precision. Furthermore, while the positive predictive value for COVID-19 low (0.12) due to its lower prevalence, this was balanced by a high negative predictive value of 0.96, which is clinically significant for ruling out the disease.

Conversely, rhinovirus and adenovirus detection presented lower AUCs (0.62 and 0.69, respectively), suggesting moderate diagnostic capability for these infections. Although their detection sensitivities were lower (0.50 and 0.57, respectively), the model achieved strong specificity (0.62 for rhinovirus and 0.69 for adenovirus), indicating that it is accurate in identifying negative cases for these viruses, particularly adenovirus. The balanced accuracy detection for these viruses (0.56 for rhinovirus and 0.63 for adenovirus) demonstrates that, while the model is less robust for the detection of these infections, it still provides valuable diagnostic information for clinical decision-making.

### 3.3. Virus-Specific Predictive Symptoms for Respiratory Infections

The analysis of virus-specific predictive clinical presentation provided insights into how different signs and symptoms correlated with the diagnosis of each respiratory virus.

**Figure 1:**
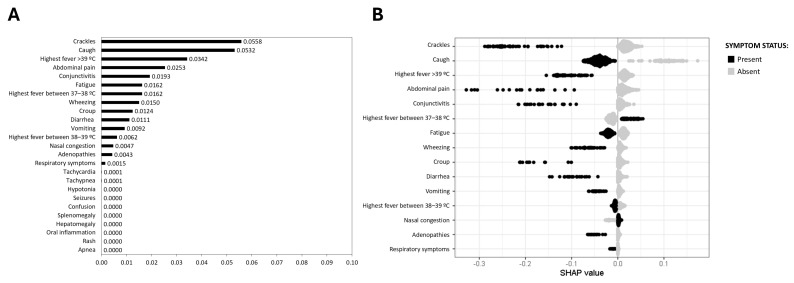
SHAP values for SARS-CoV-2 prediction. (**A**) Mean SHAP value barplot; (**B**) SHAP beeswarm plot. The black points represent the presence of a symptom associated with the disease, and the grey points represent their absence. Higher positive SHAP values indicate an increased probability of the disease, illustrating the relationship between specific symptoms and the likelihood of SARS-CoV-2 infection.

As shown in Figure 1A, the most significant symptom associated with the absence of SARS-CoV-2 infection were crackles (0.0558), cough (0.0532), fever >39 °C (0.0342), abdominal pain (0.0253), and conjunctivitis (0.0193). Other symptoms, such as wheezing (0.0150), croup (0.0124), and diarrhea (0.0111) were also linked to the absence of SARS-CoV-2 infection, but with less intensity. Interestingly, low-grade fever (37–38 °C) showed a moderate association with SARS-CoV-2 ARI (0.0162) but emerged as the key indicator among all signs and symptoms analyzed (Figure 1A,B).

**Figure 2:**
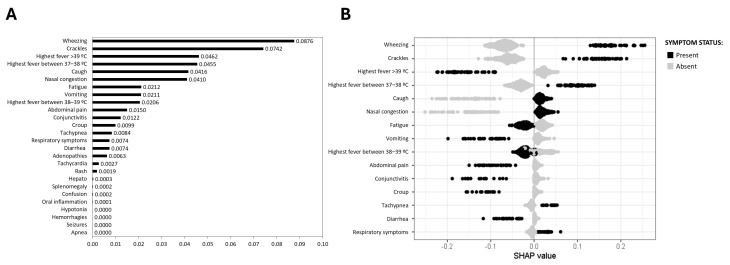
SHAP values for RSV prediction. (**A**) Mean SHAP value barplot; (**B**) SHAP beeswarm plot. The black points represent the presence of a symptom associated with the disease, and the grey points represent their absence. Higher positive SHAP values indicate an increased probability of the disease, illustrating the relationship between specific symptoms and the likelihood of RSV infection.

Wheezing (0.0876) and crackles (0.0742) emerged as the strongest predictors of RSV ARI, followed by low-grade fever (37–38 °C, 0.0455) (Figure 2A,B). In contrast, high fever (>39 °C) was the principal symptom related to the absence of RSV infection (0.0462) (Figure 2A,B).

**Figure 3:**
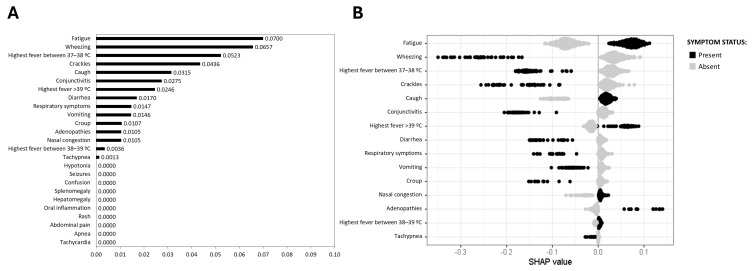
SHAP values for influenza (A + B) prediction. (**A**) Mean SHAP value barplot; (**B**) SHAP beeswarm plot. The black points represent the presence of a symptom associated with the disease, and the grey points represent their absence. Higher positive SHAP values indicate an increased probability of the disease, illustrating the relationship between specific symptoms and the likelihood of influenza (A + B) infection.

Regarding influenza (A + B), fatigue was identified as the symptom most strongly associated with an influenza diagnosis (0.0700), followed by cough (0.0315) and high fever (>39 °C) (0.0246) (Figure 3A,B). However, wheezing (0.0657), low-grade fever (37–38 °C) (0.0523), crackles (0.0436), and conjunctivitis (0.0275) were associated with the absence of influenza ARI (Figure 3A,B).

**Figure 4:**
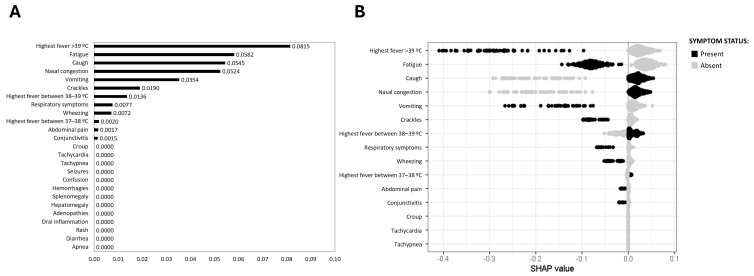
SHAP values for rhinovirus prediction. (**A**) Mean SHAP value barplot; (**B**) SHAP beeswarm plot. The black points represent the presence of a symptom associated with the disease, and the grey points represent their absence. Higher positive SHAP values indicate an increased probability of the disease, illustrating the relationship between specific symptoms and the likelihood of rhinovirus infection.

For the prediction of rhinovirus ARI, cough (0.0545), nasal congestion (0.0524), and to a lesser extent, low-grade fever (37–38 °C, 0.0020) were the most relevant symptoms (Figure 4). Conversely, high fever (>39 °C) (0.0815), fatigue (0.0582), vomiting (0.0354), and crackles (0.0190) were associated with the unlikely diagnosis of rhinovirus ARI, as showed in Figure 4A,B.

**Figure 5:**
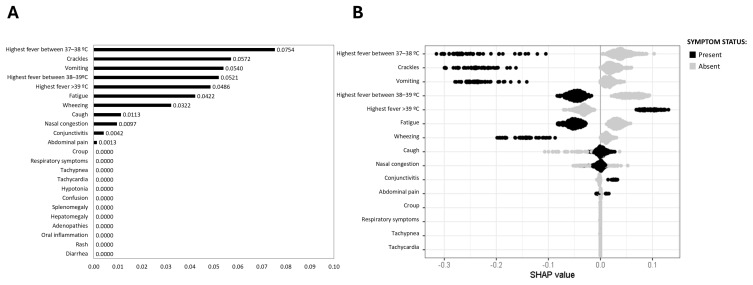
SHAP values for adenovirus prediction. (**A**) Mean SHAP value barplot; (**B**) SHAP beeswarm plot. The black points represent the presence of a symptom associated with the disease, and the grey points represent their absence. Higher positive SHAP values indicate an increased probability of the disease, illustrating the relationship between specific symptoms and the likelihood of adenovirus infection.

Adenovirus symptom analysis revealed that high fever (>39 °C) was the only symptom that exhibited a high SHAP value (0.0486), and a significant number of patients showed this symptom, as indicated by the high density of black dots (Figure 5). Strikingly, the model stands out by its predictive potential in terms of ruling out the virus presence, as reported by the connection between fever (37–38 °C) (0.0754), crackles (0.0572), vomiting (0.0540), fever (38–39 °C) (0.0521), fatigue (0.0422), and wheezing (0.0322) high SHAP values and the absence of adenovirus (Figure 5).

## 4. Discussion

This study highlights the utility of symptom-based predictive models in pediatric primary care for diagnosing specific respiratory viral infections, with the potential of facilitating their targeted management and overall improving clinical outcomes [9,13]. Such models are especially valuable during outbreaks or in novel virus scenarios, where a rapid symptom assessment can guide timely interventions and reduce reliance on confirmatory tests [15].

In this context, integrating a symptom-based predictive model into routine clinical practice appears highly promising [9]. In pediatric populations, where symptoms are often ambiguous and may evolve rapidly [9], such a tool can support healthcare providers, mainly in primary care centers, in making prompt, data-driven decisions [16]. Given that ARIs account for 50% of pediatric consultations and result in 1.2 million hospital admissions annually [17], the high rate of hospitalizations and intensive care demands for these patients impose a substantial economic burden on healthcare systems. The development of predictive models focused on providing early and accurate diagnoses is therefore crucial, as they can enhance disease management, reduce costs, and improve patient care [13]. Our model addresses this need by enabling the timely identification of infections and demonstrating notable accuracy in detecting key pathogens responsible for ARIs in children, especially in settings where access to highly sensitive or rapid diagnostic tests is limited.

Remarkably, the model demonstrated effectiveness in identifying infections not only through specific signs and symptoms but also by recognizing the lack of certain symptoms, an aspect that is equally valuable for clinical decision-making. For RSV, the model achieved an AUC of 0.81, with sensitivity of 0.64 and specificity of 0.77, showing a strong balance. This suggests that it effectively detects RSV when symptoms like wheezing or lack of high fever are present. The high AUC reflects the model’s capability to distinguish the RSV infection from other viral infections based on symptom patterns, offering reliable diagnostic utility in primary care settings. Additionally, the model displayed solid accuracy in detecting influenza, achieving an AUC of 0.70, further demonstrating its effectiveness in identifying common pathogens causing ARIs. In SARS-CoV-2 infections, the absence of symptoms such as crackles or wheezing was found to be a significant predictor of non-infection.

The model’s performance was also influenced by the clinical presentation of certain viruses, with better predictive capabilities for those with more robust manifestations, such as enveloped viruses like influenza viruses, SARS-CoV-2, and RSV, which affect both the upper and the lower respiratory tract [15,17]. These viruses are often associated with more severe clinical outcomes, such as those related to pneumopathies [18,19], and the model demonstrated particular accuracy in detecting these viruses due to their distinct clinical signatures.

The ability of our predictive model to accurately detect viruses that cause particularly severe symptoms is a significant achievement, as infections by these pathogens have higher economic costs than milder infections. This is especially relevant for RSV, which is associated with annual hospitalization expenses of up to EUR 87.1 million just in Spain [20]. Additionally, the accurate detection of viral infections is particularly relevant, as the emergence of SARS-CoV-2 has not only increased healthcare system costs but also disrupted the seasonal patterns of respiratory pathogens, making the diagnosis of viral ARIs challenging [21,22].

Conversely, non-enveloped viruses like rhinovirus and adenovirus exhibited lower AUC values of 0.62 and 0.69, respectively. These results reflect the fact that these non-enveloped viruses tend to cause milder, more localized infections, often confined to the upper respiratory tract [23,24]. Their symptoms are less specific, typically presenting as nasal congestion, pharyngitis, and cough [24,25]. As a result, the model displayed lower diagnostic accuracy for this group of viruses, relying on the absence of severe symptoms, such as high fever or systemic manifestations, to make predictions. By assessing both specific and nonspecific symptoms, the model provides clinicians with valuable insights for tailoring more effective interventions [26,27].

Building on this foundation, this predictive model demonstrates significant potential for the early diagnosis of pediatric respiratory viral infections by analyzing symptom patterns. Its dual approach—leveraging both the presence and the absence of symptoms—enhances the diagnostic accuracy for infections like those caused by SARS-CoV-2, RSV, and influenza viruses, which often require urgent intervention. This capability becomes even more important when considering future emergent viruses, where symptomatology will initially be the primary tool for diagnosis before specific tests are developed and deployed, as was seen during the initial stages of the COVID-19 pandemic. Moreover, the model’s application during high-demand periods could mitigate the healthcare system overload by reducing unnecessary testing and hospitalization.

However, the model also presents limitations, such as reduced accuracy for the detection of viral infections with mild symptoms, like rhinovirus or adenovirus infections, due to their nonspecific clinical presentations. Its performance may also have been limited by the sample size analyzed, preventing the model from offering accurate predictions across different age groups. The model also depends on accurate symptom reporting, which can vary due to clinician judgment and patient descriptions, especially in pediatric cases, potentially affecting its predictive accuracy. In addition, the model did not consider the possibility of viral co-detections, which may have been associated with overlapping signs and symptoms. Thus, while the model supports clinical decision-making, confirmatory testing remains necessary to ensure accurate diagnoses.

## 5. Conclusions

The model effectively balances sensitivity and specificity for pathogens like SARS-CoV-2, RSV, and influenza viruses, making it a valuable diagnostic tool for pediatric care. Its adaptability to different clinical environments, including resource-limited primary care settings, further underscores its practical utility. Additionally, the model’s reliance on readily available clinical data makes it an accessible and scalable solution for improving diagnostic accuracy. Its incorporation into routine practice could not only strengthen healthcare responses but also contribute to improved readiness for future pandemics.

## Figures and Tables

**Table 1:** Parameters evaluated by the model for each specific viral infection. All the values are indicated as a percentage over 1, except for the first line, which reports the number of samples for each virus.

Metric	SARS-CoV-2	RSV	Influenza	Rhinovirus	Adenovirus
Number of Samples	95	154	382	103	126
AUC	0.71	0.81	0.70	0.62	0.69
Accuracy	0.64	0.76	0.63	0.61	0.68
Kappa	0.09	0.27	0.27	0.04	0.13
Sensitivity	0.64	0.64	0.70	0.50	0.57
Specificity	0.64	0.77	0.59	0.62	0.69
Positive Predicted Value	0.12	0.29	0.50	0.09	0.17
Negative Predicted Value	0.96	0.94	0.77	0.94	0.94
Prevalence	0.08	0.13	0.37	0.07	0.10
Detection Rate	0.05	0.08	0.26	0.03	0.06
Detection Prevalence	0.38	0.28	0.52	0.39	0.33
Balanced Accuracy	0.64	0.71	0.65	0.56	0.63

## Data Availability

The datasets created and/or analyzed in this study can be obtained from the corresponding author upon reasonable request.

## Supplementary Materials

The following supporting information can be downloaded at: https://www.mdpi.com/article/10.3390/v17040546/s1, Table S1: Dataset of demographic characteristics, respiratory viral diagnoses, and symptoms in the study population; Figure S1: Percentage of missing values for each symptom; Figure S2: Co-infection correlations for the different respiratory viruses; Comic entitled “Virus identified! Why do I have such difficulty breathing?”.

## Abbreviations

The following abbreviations are used in this manuscript:

AUC	Area Under the Curve
ARI	Acute Respiratory Infection
CAP	Centre d’Atenció Primària (Primary Care Center)
COPEDICAT	Coronavirus Pediatria Catalunya
EAP	Equip d’Atenció Primària (Primary Care Team)
PCR	Polymerase Chain Reaction
RSV	Respiratory Syncytial Virus
SHAP	Shapley Additive Explanations
SMOTE-NC	Synthetic Minority Over-Sampling Technique for Nominal and Continuous Features
SARS-CoV-2	Severe Acute Respiratory Syndrome Coronavirus 2
