# Supplementary material for: Implementing Symptom-Based Predictive Models for Early Diagnosis of Pediatric Respiratory Viral Infections
Source: Viruses. 2025 Apr 8;17(4):546. doi: 10.3390/v17040546 (PMC12031125; doi:10.3390/v17040546)

Supplementary materials

Table S1. Dataset of demographic characteristics, respiratory viral diagnoses, and symptoms in the study population.

Variable	Adenovirus	Flu (A+B)	Flu A	Flu B	Rhinovirus	SARS-CoV-2	Respiratory Syncytial Virus
**Demographics**							
Age (years), median (IQR)	3 (5)	7 (7)	6 (8)	7 (6)	2 (5.5)	1 (8)	1 (1.75)
Male, n (%)	73 (57.48%)	205 (53.25%)	125 (52.30%)	82 (54.67%)	55 (53.40%)	56 (58.95%)	75 (48.70%)
Female, n (%)	53 (41.73%)	177 (45.97%)	111 (46.44%)	68 (45.33%)	48 (46.60%)	39 (41.05%)	79 (51.30%)
**Symptoms**							
Abdominal pain, n (%)	17 (13.39%)	39 (10.13%)	21 (8.79%)	18 (12%)	6 (5.83%)	4 (4.21%)	6 (3.90%)
Abnormal lung sounds, n (%)	15 (11.90%)	29 (7.67%)	21 (8.90%)	8 (5.48%)	30 (29.13%)	11 (12.22%)	98 (63.64%)
Adenopathies, n (%)	8 (6.30%)	34 (8.83%)	17 (7.11%)	17 (11.33%)	2 (1.94%)	0 (0%)	3 (1.95%)
Apnea, n (%)	0 (0%)	0 (0%)	0 (0%)	0 (0%)	0 (0%)	0 (0%)	1 (0.67%)
Conjunctivitis, n (%)	29 (22.83%)	22 (5.71%)	8 (3.35%)	14 (9.33%)	9 (8.74%)	4 (4.21%)	13 (8.44%)
Cough, n (%)	95 (74.80%)	322 (83.64%)	192 (80.33%)	134 (89.33%)	90 (87.38%)	62 (65.26%)	148 (96.10%)
Crackles, n (%)	8 (6.35%)	22 (5.82%)	16 (6.78%)	6 (4.11%)	19 (18.45%)	7 (7.78%)	74 (48.05%)
Croup, n (%)	7 (5.51%)	13 (3.38%)	8 (3.35%)	5 (3.33%)	11 (10.68%)	3 (3.16%)	11 (7.14%)
Dermatologic symptoms, n (%)	2 (1.57%)	9 (2.34%)	6 (2.51%)	3 (2%)	5 (4.85%)	3 (3.16%)	2 (1.30%)
Diarrhea, n (%)	14 (11.02%)	25 (6.49%)	12 (5.02%)	13 (8.67%)	5 (4.85%)	2 (2.11%)	12 (7.79%)
Fatigue, n (%)	50 (39.37%)	233 (60.52%)	144 (60.25%)	92 (61.33%)	30 (29.13%)	37 (38.95%)	69 (44.81%)
Fever, n (%)	123 (96.85%)	382 (99.22%)	238 (99.58%)	148 (98.67%)	78 (75.73%)	87 (91.58%)	121 (78.57%)
Gastrointestinal symptoms, n (%)	37 (29.13%)	79 (20.52%)	40 (16.74%)	40 (26.67%)	14 (13.59%)	15 (15.79%)	20 (12.99%)
General coinfection, n (%)	37 (29.13%)	33 (8.57%)	14 (5.86%)	19 (12.67%)	48 (46.60%)	23 (24.21%)	36 (23.38%)

Headache, n (%)	26 (25.24%)	173 (49.86%)	101 (47.20%)	74 (54.41%)	19 (20.43%)	18 (25.35%)	14 (11.86%)
Hemorrhages, n (%)	0 (0%)	0 (0%)	0 (0%)	0 (0%)	0 (0%)	0 (0%)	1 (0.65%)
Hepatomegaly, n (%)	4 (3.15%)	0 (0%)	0 (0%)	0 (0%)	0 (0%)	1 (1.05%)	0 (0%)
Highest fever between 37-38ºC, n (%)	14 (11.38%)	46 (12.11%)	31 (13.08%)	16 (10.88%)	31 (40.26%)	33 (38.37%)	39 (32.50%)
Highest fever between 38-39ºC, n (%)	60 (48.78%)	239 (62.89%)	147 (62.03%)	93 (63.27%)	37 (48.05%)	42 (48.84%)	64 (53.33%)
Highest fever over 39ºC, n (%)	49 (39.84%)	95 (25%)	59 (24.89%)	38 (25.85%)	9 (11.69%)	11 (12.79%)	17 (14.17%)
Hypotonia, n (%)	0 (0%)	1 (0.26%)	0 (0%)	1 (0.67%)	0 (0%)	0 (0%)	0 (0%)
Loss of smell, n (%)	0 (0%)	0 (0%)	0 (0%)	0 (0%)	2 (2.41%)	0 (0%)	1 (1.12%)
Loss of taste, n (%)	1 (1.09%)	0 (0%)	0 (0%)	0 (0%)	0 (0%)	0 (0%)	0 (0%)
Nasal congestion, n (%)	100 (78.74%)	314 (81.56%)	187 (78.24%)	130 (86.67%)	88 (85.44%)	69 (72.63%)	140 (90.91%)
Neurological symptoms, n (%)	0 (0%)	4 (1.04%)	3 (1.26%)	1 (0.67%)	0 (0%)	0 (0%)	2 (1.30%)
Nuchal stiffness, n (%)	0 (0%)	0 (0%)	0 (0%)	0 (0%)	0 (0%)	0 (0%)	0 (0%)
Odynophagia, n (%)	39 (30.71%)	163 (42.34%)	104 (43.51%)	60 (40%)	26 (25.24%)	23 (24.21%)	38 (24.68%)
Oral inflammation, n (%)	0 (0%)	2 (0.52%)	2 (0.84%)	0 (0%)	0 (0%)	1 (1.05%)	0 (0%)
Peripheral paralysis, n (%)	0 (0%)	0 (0%)	0 (0%)	0 (0%)	0 (0%)	0 (0%)	0 (0%)
Rash, n (%)	2 (1.57%)	6 (1.56%)	4 (1.67%)	2 (1.33%)	5 (4.85%)	2 (2.11%)	1 (0.65%)
Respiratory distress (mild), n (%)	7 (100%)	7 (70%)	3 (60%)	4 (80%)	11 (78.57%)	3 (60%)	29 (54.72%)
Respiratory distress (moderate), n (%)	0 (0%)	3 (30%)	2 (40%)	1 (20%)	3 (21.43%)	2 (40%)	20 (37.74%)
Respiratory distress (severe), n (%)	0 (0%)	0 (0%)	0 (0%)	0 (0%)	0 (0%)	0 (0%)	4 (7.55%)
Seizures, n (%)	0 (0%)	1 (0.26%)	1 (0.42%)	0 (0%)	0 (0%)	0 (0%)	1 (0.65%)
Shock, n (%)	0 (0%)	0 (0%)	0 (0%)	0 (0%)	0 (0%)	0 (0%)	0 (0%)
Splenomegaly, n (%)	3 (2.36%)	0 (0%)	0 (0%)	0 (0%)	0 (0%)	0 (0%)	0 (0%)
Subset coinfection, n (%)	32 (25.20%)	21 (5.45%)	8 (3.35%)	13 (8.67%)	35 (33.98%)	20 (21.05%)	31 (20.13%)
Tachycardia, n (%)	3 (2.36%)	3 (0.78%)	1 (0.42%)	2 (1.33%)	3 (2.91%)	0 (0%)	20 (12.99%)
Tachypnea, n (%)	5 (3.94%)	9 (2.34%)	6 (2.51%)	3 (2%)	4 (3.88%)	3 (3.16%)	33 (21.43%)
Vomiting, n (%)	24 (18.9%)	52 (13.51%)	30 (12.55%)	23 (15.33%)	12 (11.65%)	14 (14.74%)	12 (7.79%)
Wheezing, n (%)	9 (7.14%)	15 (3.97%)	12 (5.08%)	3 (2.05%)	23 (22.33%)	10 (11.11%)	73 (47.40%)

Figure S1. Percentage of missing values for each symptom.

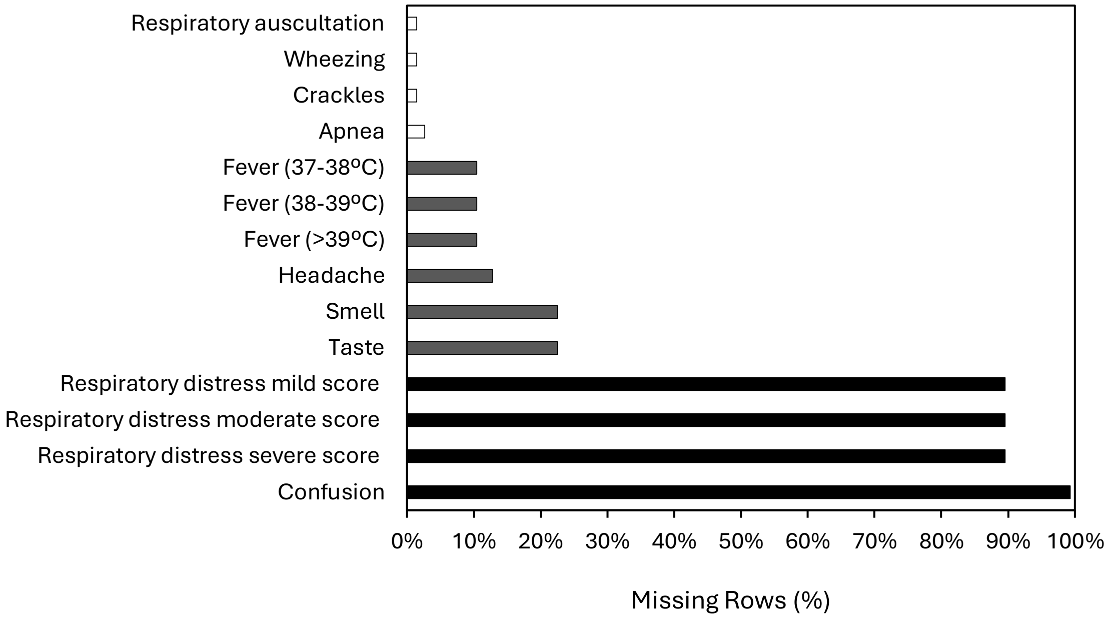

Figure S2. Co-infection correlation among the different respiratory viruses.

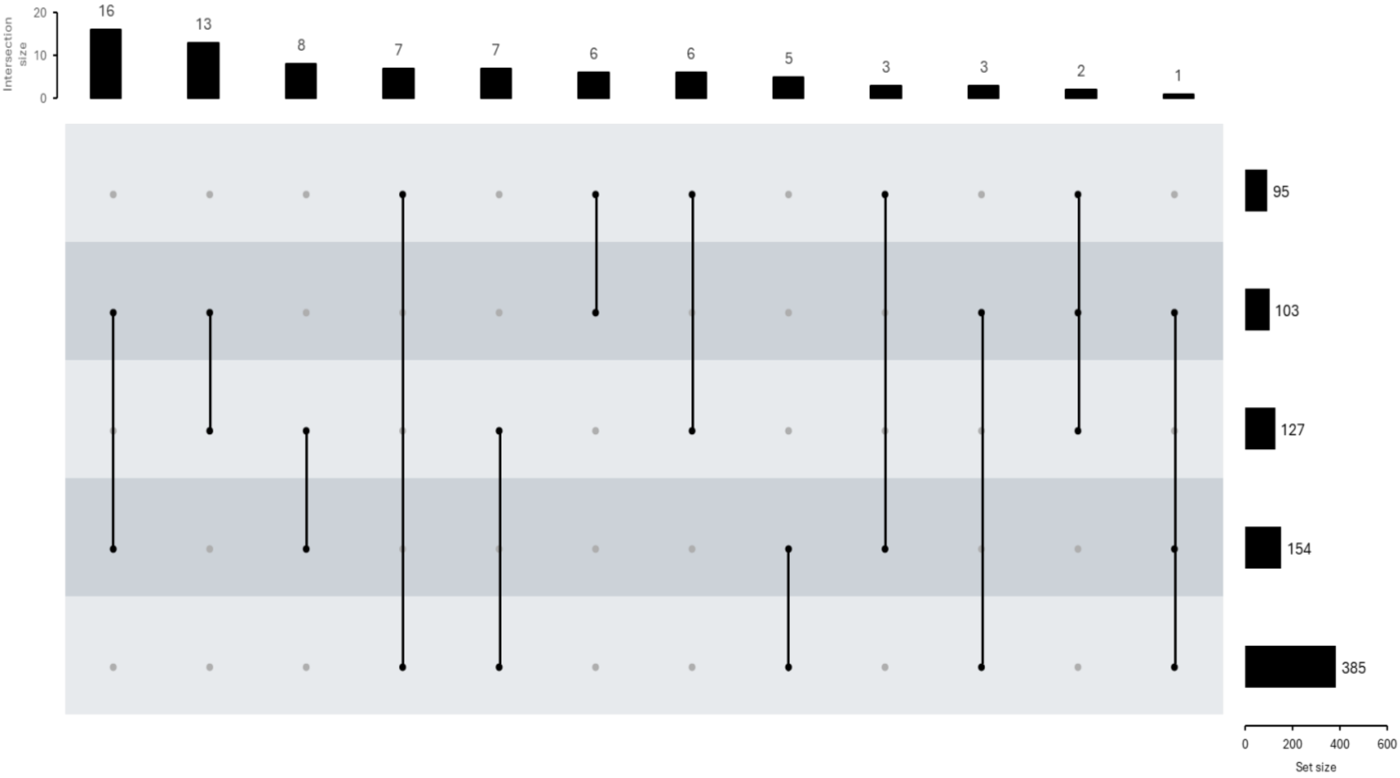

Supplement: Supplementary file 1 [file viruses-17-00546-s001.zip › viruses-3539168-supplementary.pdf]
